# Long-Term Safety of Bone Regeneration Using Autologous Stromal Vascular Fraction and Calcium Phosphate Ceramics: A 10-Year Prospective Cohort Study

**DOI:** 10.1093/stcltm/szad045

**Published:** 2023-08-01

**Authors:** Vivian Wu, Jenneke Klein-Nulend, Nathalie Bravenboer, Christiaan M ten Bruggenkate, Marco N Helder, Engelbert A J M Schulten

**Affiliations:** Department of Oral Cell Biology, Academic Centre for Dentistry Amsterdam (ACTA), University of Amsterdam and Vrije Universiteit Amsterdam, Amsterdam Movement Sciences, Amsterdam, The Netherlands; Department of Oral and Maxillofacial Surgery/Oral Pathology, Amsterdam UMC and Academic Centre for Dentistry Amsterdam (ACTA), Vrije Universiteit Amsterdam, Amsterdam Movement Sciences, Amsterdam, The Netherlands; Department of Oral Cell Biology, Academic Centre for Dentistry Amsterdam (ACTA), University of Amsterdam and Vrije Universiteit Amsterdam, Amsterdam Movement Sciences, Amsterdam, The Netherlands; Department of Clinical Chemistry, Amsterdam UMC, Vrije Universiteit Amsterdam, Amsterdam Movement Sciences, Amsterdam, The Netherlands; Department of Oral and Maxillofacial Surgery/Oral Pathology, Amsterdam UMC and Academic Centre for Dentistry Amsterdam (ACTA), Vrije Universiteit Amsterdam, Amsterdam Movement Sciences, Amsterdam, The Netherlands; Department of Oral and Maxillofacial Surgery/Oral Pathology, Amsterdam UMC and Academic Centre for Dentistry Amsterdam (ACTA), Vrije Universiteit Amsterdam, Amsterdam Movement Sciences, Amsterdam, The Netherlands; Department of Oral and Maxillofacial Surgery/Oral Pathology, Amsterdam UMC and Academic Centre for Dentistry Amsterdam (ACTA), Vrije Universiteit Amsterdam, Amsterdam Movement Sciences, Amsterdam, The Netherlands

**Keywords:** bone regeneration, calcium phosphates, dental implants, safety, sinus floor augmentation, stromal vascular fraction

## Abstract

This prospective cohort study aimed to assess long-term safety, dental implant survival, and clinical and radiological outcomes after maxillary sinus floor elevation (MSFE; lateral window technique) using freshly isolated autologous stromal vascular fraction (SVF) combined with calcium phosphate ceramics. All 10 patients previously participating in a phase I trial were included in a 10-year follow-up. They received either β-tricalcium phosphate (β-TCP; *n* = 5) or biphasic calcium phosphate (BCP; *n* = 5) with SVF-supplementation on one side (study). Bilaterally treated patients (6 of 10; 3 β-TCP, 3 BCP) received only calcium phosphate on the opposite side (control). Clinical and radiological assessments were performed on 44 dental implants at 1-month pre-MSFE, and 0.5- to 10-year post-MSFE. Implants were placed 6 months post-MSFE. No adverse events or pathology was reported during a 10-year follow-up. Forty-three dental implants (98%) remained functional. Control and study sides showed similar peri-implant soft-tissue quality, sulcus bleeding index, probing depth, plaque index, keratinized mucosa width, as well as marginal bone loss (0-6 mm), graft height loss (0-6 mm), and graft volume reduction. Peri-implantitis was observed around 6 implants (control: 4; study: 2) in 3 patients. This study is the first to demonstrate the 10-year safety of SVF-supplementation in MSFE for jawbone reconstruction. SVF-supplementation showed enhanced bone regeneration in the short term (previous study) and led to no abnormalities clinically and radiologically in the long term.

## Graphical Abstract

**Graphical Abstract:**
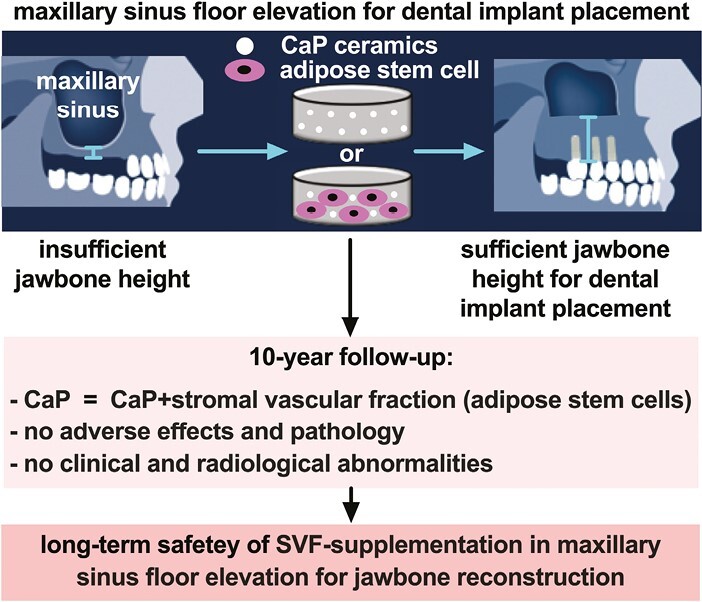

**Significance Statement**

Ten patients received freshly isolated autologous stromal vascular fraction (SVF)-supplementation containing adipose stromal/stem cells, in maxillary sinus floor elevation (MSFE) using calcium phosphate ceramics to allow dental implant placement. Short-term feasibility, safety, and potential efficacy of SVF-supplementation for bone regeneration have been previously demonstrated in a phase I clinical trial. SVF-supplementation resulted in no abnormalities clinically and radiologically after a 10-year follow-up demonstrating long-term safety. SVF-supplementation in MSFE might improve dental implant success, and open new possibilities for cell-based bone tissue engineering applications.

## Introduction

Bone regeneration to restore bone defects in the oral and maxillofacial region remains challenging. Patients with insufficient alveolar bone height in the lateral maxilla for dental implant placement are currently treated with maxillary sinus floor elevation (MSFE), using autologous bone and/or bone substitute.^1^ Autologous bone is considered the gold standard grafting material in MSFE, due to the essential combination of osteogenic, osteoinductive, and osteoconductive properties as well as prevention of immunogenic responses.^2^ However, morbidity caused by the harvesting procedure and limited availability of autologous bone encourage the search for suitable alternatives with similar bioactivity.

Calcium phosphate ceramics show low bone in-growth rates in comparison with autologous bone grafts since they only have osteoconductive properties and lack osteoinductive potential.^3^ Many studies have actively been conducted to improve the bioactivity of calcium phosphate ceramics in bone regeneration (for a review, see: Jeong et al).^4^ Cell-based bone tissue engineering is a promising strategy to improve bone formation by providing osteogenic cells that secrete osteoinductive signals to recruit cells from surrounding bone.^5-8^ This might shorten the time needed to obtain sufficient bone regeneration than is required when using bone substitute only.

During the past decades, multiple sources of stem cells have been investigated for bone tissue engineering in the oral and maxillofacial region.^8^ Adipose tissue-derived mesenchymal stem cells (ASCs) have opened new possibilities in adult stem cell therapies, since the use of ASCs has eliminated drawbacks associated with adult bone marrow-derived mesenchymal stem cells (eg, costly and time-consuming Good Manufacturing Practice (GMP)-expansion necessary, painful harvesting procedure), which are still the most frequently used cells in cell-based bone tissue engineering.^8^ The stromal vascular fraction (SVF) of human adipose tissue is considered a promising source for essential cells with osteogenic and angiogenic potential.^7-11^ Moreover, SVF is a cell source with clinical feasibility due to the large quantities that can be harvested and applied in a one-step surgical procedure.^7,12^ A disadvantage of SVF harvesting so far is that liposuction of adipose tissue is performed under general anesthesia and requires (short) hospitalization.

Clinical evidence of ASC-application for bone regeneration is limited, although the potential of ASCs evokes high expectations.^7,13-15^ To the best of our knowledge, only one study reported unsatisfactory clinical results of ASC-application in cranioplasty during a 6-year follow-up; 4 out of patients suffered from unsatisfactory treatment outcome partially due to poor ossification, infection, or tumor recurrence, and 2 patients had to be re-operated due to graft resorption.^16^ Stem cell-application in regenerative medicine has also raised safety concerns, eg, tumorigenic potential and biodistribution.^17^ Therefore, clinical studies investigating long-term safety and efficacy are essential before continuing with clinical applications using (adipose) stem cells.

In a previous phase I clinical trial, 10 patients underwent MSFE prior to dental implant placement using freshly isolated autologous SVF seeded on either β-tricalcium phosphate (β-TCP) or biphasic calcium phosphate (BCP) carriers in a one-step surgical procedure.^7^ No serious adverse events were reported during the procedure and 3-year follow-up. In addition, biopsies showed more induction of bone mass and bone formation by SVF-supplementation compared to the calcium phosphate carriers, in particular in β-TCP-treated patients.^7^ Paired analysis of the 6 bilaterally treated patients revealed markedly higher bone volume and bone formation, demonstrating an additive effect of SVF, independent of the bone substitute.^7^ Moreover, more bone mass seemed to correlate with blood vessel formation, and was higher in the cranial part of the study biopsies, in particular in ­β-TCP-treated patients.^9^ Based on short-term results (~3 years), we demonstrated for the first time the feasibility, safety, and potential efficacy of SVF seeded on calcium phosphate carriers, and indicated a pro-angiogenic effect of SVF.^7,9^

The present prospective cohort study was designed as a 10-year follow-up of patients previously enrolled in our phase I clinical trial.^7^ We aimed to assess long-term safety, dental implant survival, and clinical and radiological outcomes after MSFE (lateral window technique) using calcium phosphate ceramics (β-TCP or BCP) with and without freshly isolated autologous SVF-supplementation.

## Materials and Methods

### Study Design and Patient Selection

This prospective cohort study was designed as a 10-year follow-­up of patients previously enrolled in a phase I clinical trial investigating safety and potential additive effect of SVF in MSFE using lateral window technique on bone regeneration.^7^ Ten patients who required dental implants for prosthetic rehabilitation in the partially edentulous posterior maxilla were included. All patients had an adequate alveolar bone height of at least 4 mm, but not more than 8 mm at the lateral maxilla. Detailed patient inclusion and exclusion criteria have been previously described.^7^ Firstly, 4 patients with unilateral MSFE-indication, and 6 patients with bilateral MSFE-indication were selected by consecutive sampling.^7^ Secondly, 2 equal treatment groups were created by random treatment allocation, ie, β-TCP (5 patients: 2 unilateral, 3 bilateral) or BCP-treatment (5 patients: 2 unilateral, 3 bilateral) group. All unilateral MSFE-patients received calcium phosphate with SVF. All bilateral MSFE-patients (split-mouth) received pure calcium phosphate on one side (control), and calcium phosphate with SVF on the other side of the maxilla (study). Control and study sides were randomly assigned to prevent bias. The surgical procedures have been previously described.^7^ In short, adipose tissue was processed with a CE-marked Celution device (Cytori Therapeutics, Inc., San Diego, CA, USA) to obtain SVF. For implantation, scaffolds were seeded with 10^7^ nucleated SVF-cells (~2 × 10^5^ ASC-like cells)/g calcium phosphate carrier (Ceros β-TCP (Thommen Medical, Grenchen, Switzerland) or Straumann Bone Ceramic consisting of 60% hydroxyapatite (HA) and 40% β-TCP (Straumann AG, Basel, Switzerland)). Implants were placed 6-months post-MSFE in a single-stage procedure under local anesthesia. Extensive cell characterizations were performed, as described previously.^7^ In short, the SVF surface marker expression profiles as determined with fluorescence-activated cell sorting were consistent with previous reports, including the CD34 positivity reported for ASCs.^18-20^ Radiologically, all implants showed absence of bone loss beyond marginal bone-level changes resulting from initial bone remodeling at 3 months after implant placement. All implants showed peri-implant health (absence of erythema, heavy bleeding on probing, swelling, suppuration, and probing depth <6 mm) during a short-term follow-up (~3 years). All patients received dental implant maintenance in regular Dutch oral health care system, ie, 1 to 4 visits to the dentist and/or oral hygienist per year.

The present study was conducted according to the guidelines of the Declaration of Helsinki and approved by the medical ethical committee (IRB) of the VU University Medical Center in Amsterdam (Dossier number: 2020.344: ABR NL71857.029.20). All patients signed a written informed consent before participation in the study. The study was performed according to the STROBE guidelines.^21^

One experienced surgeon (VW) performed anamnesis, as well as all clinical (intra-oral) and quantitative radiological assessments. Three experienced surgeons (V.W., C.M.B., E.A.J.M.S.) carried out qualitative radiological assessments. Intra- and inter-examiner calibration was done before the start of the study. Intra-examiner calibration included identification of clinical and radiological reference points and associated radiological linear measurements. Inter-examiner calibration included identification of pathology and abnormalities. Discrepancies between examiners were resolved through discussion. Treatment groups remained concealed for examiners. Panoramic radiographs and CBCT-scans were taken at different time points during the previous phase I study^7^ and at the 10-year follow-up (Supplementary Table S1). To control for enlargement of the anatomical structures as a result of panoramic radiography, dental implant length was used as a reference.

### Primary Outcome Measure

#### Safety

Safety outcomes related to the product or procedure were assessed. General health changes and (serious) adverse events were patient reported through a health questionnaire. Maxillary sinus-related problems such as sinusitis were patient reported through anamnesis. Clinical (intra-oral) and radiological assessments (panoramic radiographs and cone beam computerized tomography (CBCT) scans) were carried out to detect any pathology and abnormalities.

### Secondary Outcome Measures

#### Implant Survival

Number of implants in situ was counted.

#### Peri-implant Condition

Soft-tissue physical appearance was scored per dental implant site as no abnormalities or swollen. Soft-tissue color was scored as white-necrotic, pink or red, and soft-tissue surface morphology at the buccal aspect of each implant as smooth or stippled.

Sulcus bleeding index (SBI) and probing depth (PD; in mm) were scored at 4 locations (mesial, buccal, distal, and palatal) around the implant. SBI was scored as score 0: no bleeding when passing a periodontal probe along the gingival margin adjacent to the implant; score 1: isolated bleeding spot; score 2: confluent red line of blood on margin; score 3: heavy or profuse bleeding.^22^ Probing depth was registered using a manual probe and light force (~0.25 N). The highest values of SBI and PD were used for statistical analyses at the patient and implant level.

Marginal bone loss (in mm) was assessed on panoramic radiographs. Marginal bone loss was defined as the perpendicular distance from the implant-abutment interface to the radiographic bone level at the mesial and distal aspects of each implant, since the implants were placed “flush” (at the same height) to the alveolar ridge. Mean values of marginal bone loss at the mesial and distal aspect of each implant were calculated, and the highest value per patient and per implant site was used for statistical analysis.

Since baseline radiographic and probing data were absent, the following case definitions of peri-implant health and disease, ie, peri-implant mucositis and peri-implantitis, were applied at the 10-year follow-up: Peri-implant health: absence of erythema, bleeding on probing, swelling, and suppuration; Peri-implant mucositis: bleeding on gentle probing <0.25 N, absence of bone loss >2 mm; Peri-implantitis: ≥3 mm marginal bone loss combined with ≥6 mm PD with bleeding and/or suppuration.^23^

#### Peri-implant Disease Risk Factors

Patient-reported smoking and diabetes were obtained through a health questionnaire. Patient-reported dental implant maintenance, and dental implant-related biological complications were obtained through anamnesis. Full-mouth periodontal condition screening was carried out according to the Dutch Periodic Periodontal Screening-index (PPS) by one examiner.^24^ Probing depth was scored at 4 locations (mesial, buccal, distal, and palatal) around every tooth. Then the highest PPS-score for each quadrant was determined: score 1: probing depth: 0-3 mm; score 2: probing depth 4-5 mm; score 3: probing depth >6 mm. Plaque index (PI) was scored by one examiner at 4 locations (mesial, buccal, distal, and palatal), as score 0: no plaque; score 1: plaque only recognized by running a probe across the smooth marginal implant surface; score 2: plaque was seen by the naked eye; score 3: soft matter abundance.^22^ The width of keratinized mucosa (KM; in mm) was scored at one location (buccal) around the dental implant. The highest PI values and the lowest KM values were used for statistical analysis at the patient and implant level. Furthermore, overcontouring (emergence angle and convexity) of the abutment-prosthesis complex was assessed clinically and radiologically.

#### Technical Complications

Patient-reported longevity of the restorations were obtained through anamnesis. Implant, connection, or suprastructure-related technical complications were assessed clinically and radiologically (panoramic radiograph).

#### Graft and Residual Maxillary Sinus Characteristics

Pathology, eg, Schneiderian mucosal hypertrophy (>1 mm), mucosal cysts, polyps, bone lesions, neoplasms, and antroliths were assessed on panoramic radiographs and CBCT scans. Irregularities of graft or residual maxillary sinus area and recognizable demarcation of the original maxillary sinus floor and radiopaque graft were assessed on CBCT scans. Graft structure was scored as homogenous or non-homogenous, and as bone like or non-bone like. Any radiopacities or radiolucencies in the grafted area, and/or graft scalloping were documented. The residual maxillary sinus area was characterized as air filled or non-air filled.

#### Tissue Height and Graft Height Loss

Tissue height (in mm) was assessed on panoramic radiographs. Tissue height was defined as the perpendicular distance from the implant-abutment interface (dental implant mid axis) to the cranial border of the graft. Graft height loss (in mm) was calculated by subtracting tissue height at the 10-year ­follow-up from tissue height at dental implant placement. Mean values of tissue height and graft height loss were calculated for study and control sides, and used for statistical analysis on patient level.

### Statistical Analysis

Data are presented as mean ± standard deviation (SD). Statistical analysis was performed with GraphPad Prism 9 software (GraphPad, La Jolla, CA, USA; http://www.graphpad.com/). Mann-Whitney *U* test was used to test differences in clinical (SBI, PD, PI, and KM) and radiological outcomes (marginal bone and graft height loss) between β-TCP or BCP study (with SVF) and control sides (without SVF), at the patient and/or implant level. Study and control sides were assumed independent variables in bilaterally treated patients. The Mann-Whitney *U test* showed no statistical differences between data from β-TCP and BCP study and control sides. Therefore, data of β-TCP and BCP-treated patients were pooled, and a (paired) Wilcoxon-signed rank test was used to test differences between study and control sides. To investigate a possible relationship between different clinical and radiological outcomes, Spearman correlation test was conducted. KruskalWallis test was performed to test differences in (baseline) alveolar bone height between β-TCP, β-TCP+SVF, BCP, and BCP+SVF-treated sides. Statistical significance was considered if *P* < .05.

## Results

### Patients Enrolled

All 10 patients who had participated in the previous phase I clinical trial were included in the 10-year follow-up (Table 1). The average patient age at a 10-year follow-up was 66 ± 7 years (range: 56-79 years). A total of 44 dental implants were included for analysis.

**Table 1.** Patient data.

Pt#	Gender (♂,♀), age (years)	Unilateral/ bilateral case	Control/study side	Graft material	Dental implant positions
1	♀, 69	Bilateral	Control	β-TCP	*14*, 15, 16
			Study	β-TCP	*24*, 25, 26
2	♀, 56	Bilateral	Control	β-TCP	*24*, 25, 26
			Study	β-TCP	14, 15, 16
3	♂, 79	Bilateral	Control	β-TCP	*14*, 15, 16
			Study	β-TCP	25, 26, 27
4	♀, 69	Unilateral	Study	β-TCP	*24*, 25, 26
5	♂, 74	Unilateral	Study	β-TCP	15, 16
6	♀, 66	Bilateral	Control	BCP	24, 26
			Study	BCP	*14*, 15, 16
7	♂, 65	Bilateral	Control	BCP	25, 26, 27
			Study	BCP	*15*, 16, 17
8	♀, 61	Unilateral	Study	BCP	14, 15, 16
9	♂, 62	Unilateral	Study	BCP	*23*, 25, 26
10	♀, 62	Bilateral	Control	BCP	25, 26
			Study	BCP	15, 16

Gender and age at time of 10-year follow-up, whether the patients were treated unilaterally or bilaterally (“split-mouth design”), bone substitute used to augment bone of the maxillary sinus floor, and the dental implant positions (Fédération Dentaire Internationale system) are given. Italicalized numbers, biopsies completely positioned in residual native bone. Pt#, patient number; β-TCP, β-tricalcium phosphate; BCP, biphasic calcium phosphate.

### Primary Outcome Measure

#### Safety

No general health changes, (serious) adverse events, pathology, or abnormalities related to the product or procedure were observed. The 10-year follow-up was uneventful for 7 out of 10 patients. Patient #3 was diagnosed with coronary artery disease, hypertension, and diabetes mellitus type II. Patient #5 was diagnosed with heart arrhythmias, hypertension, and a cerebral vascular accident. Patient #10 was diagnosed with heart arrhythmias. None of these events were related to the product or procedure. No patient-reported any complications related to dental implant treatment and/or MSFE.

### Secondary Outcome Measures

#### Implant Survival

One implant, completely positioned in alveolar bone, was replaced due to failure within 6-months after initial dental implant placement (patient #1, study side, β-TCP, implant site 24). Implant survival rate at a 10-year follow-up was 92.9% (13 out of 14) for study sides, and 100% (9 out of 9) for control sides in β-TCP-treated patients, as well as for study sides (14 out of 14) and control sides (7 out of 7) in BCP-treated patients.

#### Peri-implant Condition

**Figure 1.**
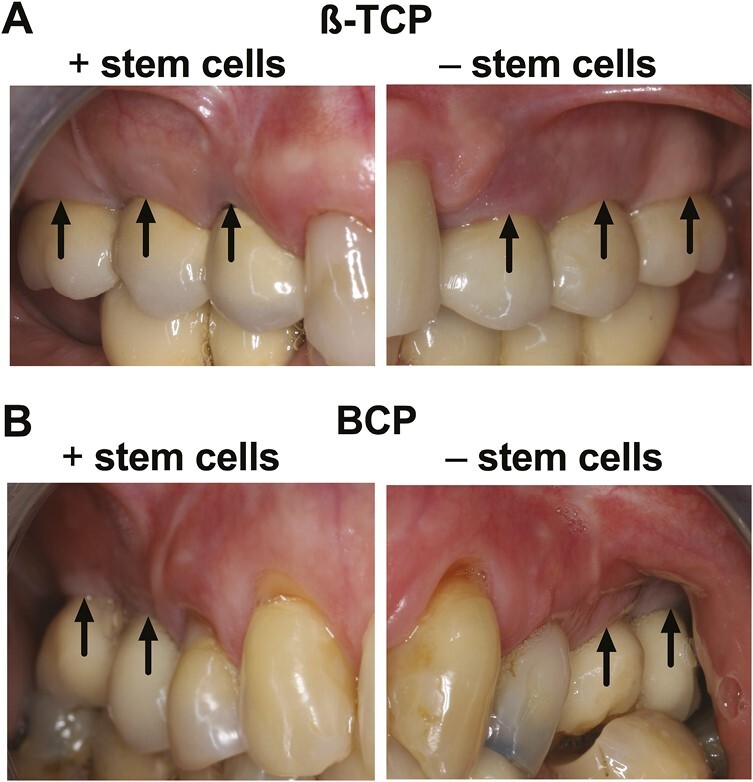
Representative intra-oral photographs of β-TCP-treated and BCP-treated patients with and without stem cells at 10 years after implant placement. (A) Intra-oral photographs of bilaterally treated patients with β-TCP plus stem cells and β-TCP only, (B) BCP plus stem cells and BCP only. Soft-tissue soft-tissue around the dental implants showed a physical appearance with no abnormalities, ie, pink colored, firm tissue quality, stippled surface morphology, and ≥2 mm width of keratinized mucosa. β-TCP, β-tricalcium phosphate; BCP, and biphasic calcium phosphate. Black arrows: implant positions.

Physical soft-tissue appearance with no abnormalities, ie,ie, pink color and stippled surface morphology, was observed (buccal) at dental implants from study and control sides of β-TCP and BCP-treated patients (Fig. 1).

At the patient level, SBI was similar between study and control sides in β-TCP-treated (*P* = .892) and BCP-treated patients (*P* = .357; Table 2). SBI was similar between study and control sides in bilaterally treated patients (*P* = .500; Table 2). PD was similar between study and control sides in β-TCP-treated (*P* = .357) and BCP-treated patients (*P* = .357; Table 2). PD was similar between study and control sides in bilaterally treated patients (*P* = .125; Table 2). Marginal bone loss was similar between study and control sides in ­β-TCP-treated (*P* > .999) and BCP-treated patients (*P* = .732; Table 2). Marginal bone loss was similar between study and control sides in bilaterally treated patients (*P* = .250; Table 2). Clinical and radiological outcomes of SVF-supplementation at the site level are summarized in Supplementary Tables S2 and S4.

**Table 2.** Clinical (sulcus bleeding index, probing depth, plaque index, width of keratinized mucosa), and radiological (marginal bone loss) outcomes of β-TCP and BCP with and without SVF-supplementation at the patient level at 10-year follow-up.

	Patient level (#sides)								
	β-TCP			BCP			β-TCP or BCP		
	Unilateral (2 patients) Bilateral (3 patients)			Unilateral (2 patients) Bilateral (3 patients)			Bilateral (6 patients)		
	Control side	Study side		Control side	Study side		Control side	Study side	
	− stem cells (*n* = 3)	+ stem cells (*n* = 5)	*P*	− stem cells (*n* = 3)	+ stem cells (*n* = 5)	*P*	- stem cells (*n* = 6)	+ stem cells (*n* = 6)	*P*
Sulcus bleeding index (SBI)									
0	0	0	.892	0	0	.357	0	0	.500
1	1	3		1	3		2	3	
2	2	1		1	2		3	3	
3	0	1		1	0		1	0	
Probing depth (PD; mm)									
0-3	0	0	.357	0	0	.375	0	0	.125
4-5	1	3		1	4		2	4	
6-7	1	2		1	1		2	2	
8-9	1	0		1	0		2	0	
Plaque index (PI)									
0	3	4	>.999	1	2	>.999	4	4	>.999
1	0	1		2	2		2	1	
2	0	0		0	1		0	1	
3	0	0		0	0		0	0	
Width keratinized mucosa (KM; mm)									
≥ 2	3	4	>.999	2	3	>.999	5	3	.500
0 - 1	0	1		1	2		1	3	
Marginal bone loss (mm)									
0-2	1	3	>.999	2	4	.732	3	5	.250
3-4	2	2		0	1		2	1	
5-6	0	0		1	0		1	0	

For each patient the highest value of sulcus bleeding index, probing depth, plaque index, and marginal bone loss was scored, as well as the lowest value of width of keratinized mucosa. *Study side significantly different from control side, *P* < .05. β-TCP, β-tricalcium phosphate; BCP, biphasic calcium phosphates; *n*, number of patients.

At the implant level, SBI was similar between study and control sides in β-TCP-treated (*P* = .405) and BCP-treated patients (*P* = .092; Table 3). SBI was similar between study and control sides in bilaterally treated patients (*P* > .999; Table 3). PD was also similar between study and control sides in β-TCP-treated (*P* = .250) and BCP-treated patients (*P* = .677; Table 3). PD was similar between study and control sides in bilaterally treated patients (*P* = .343; Table 3). Marginal bone loss was similar between study and control sides in β-TCP-treated (*P* = .162) and BCP-treated patients (*P* = .767; Table 3). Marginal bone loss was similar between study and control sides in bilaterally treated patients (*P* = .188; Table 3).

**Table 3:** Clinical (sulcus bleeding index, probing depth, plaque index, width of keratinized mucosa) and radiological (marginal bone loss) outcomes of SVF-supplementation at the implant level at 10-year follow-up.

	Implant level (#dental implants)								
	β-TCP			BCP			β-TCP or BCP		
	Unilateral (2 patients) Bilateral (3 patients)			Unilateral (2 patients) Bilateral (3 patients)			Bilateral (6 patients)		
	Control Side	Study side		Control side	Study side		Control side	Study side	
	− stem cells (*n* = 9)	+ stem cells (*n* = 14)	*P*	− stem cells (*n* = 7)	+ stem cells (*n* = 14)	*P*	stem cells (*n* = 16)	+ stem cells (*n* = 17)	*P*
Sulcus bleeding index (SBI)									
0	2	1	.405	0	2	.092	2	1	.146
1	1	9		4	10		5	13	
2	6	3		2	2		8	3	
3	0	1		1	0		1	0	
Probing depth (PD; mm)									
0-3	0	1	.250	1	0	.677	1	1	.343
4-5	5	10		4	12		9	13	
6-7	4	3		1	2		5	3	
8-9	0	0		1	0		1	0	
Plaque index (PI)									
0	9	11	.240	4	7	.898	13	13	>.999
1	0	3		3	6		3	3	
2	0	0		0	1		0	1	
3	0	0		0	0		0	0	
Width keratinized mucosa (KM; mm)									
≥2	9	13	>.999	6	11	>.999	15	12	.218
0-1	0	1		1	3		1	5	
Marginal bone loss (mm)									
0-2	5	12	.162	6	13	.767	11	16	.188
3-4	4	2		0	1		4	1	
5-6	0	0		1	0		1	0	

For each implant, the highest value of sulcus bleeding index, probing depth, plaque index, marginal bone loss was scored, as well as the lowest value of width of keratinized mucosa. *Study side significantly different from control side, *P* < .05. β-TCP, β-tricalcium phosphate; BCP, biphasic calcium phosphate; n, number of implants.

Peri-implant health was observed around 2 dental implants at the control side of one β-TCP-treated patient (pt#3), as well as around 1 dental implant at the study side of one BCP-treated patient (pt#9; Supplementary Table S5). At the control side, peri-implantitis was observed around 3 dental implants of 1 β-TCP-treated patient (pt#1), and around 1 implant of 1 BCP-treated patient (pt#7; Supplementary Table S5). At the study side, peri-implantitis was observed around 1 dental implant of 1 β-TCP-treated patient (pt#4), and around 1 implant of 1 BCP-treated patient (pt#7; Supplementary Table S5). Peri-mucositis was observed around all other implants at control and study sides (Supplementary Table S5).

#### Peri-implant Disease Risk Factors

All patients were nonsmokers. Patient #3 was diagnosed with diabetes mellitus type II. Regular dental implant maintenance program (1-4 times/year) was carried out in 9 out of 10 patients by the dentist and/or oral hygienist. Patient #7 reported no dental implant maintenance program for 2 years. No patient-reported biological complications were registered. Periodontal condition screening revealed a PPS score of 1 (0-3 mm) or 2 (4-5 mm) in all patients.

At the patient level, PI at dental implant sites was similar between study and control sides in β-TCP-treated (*P* > .999) and BCP-treated patients (*P* > .999; Table 2). PI was similar between study and control sides in bilaterally treated patients (*P* > .999; Table 2). KM was similar in study and control sides in β-TCP-treated (*P > .*999) and BCP-treated patients (*P* > .999; Table 2). KM was similar between study and control sides in bilaterally treated patients (*P* = .500; Table 2). Clinical and radiological outcomes of SVF-supplementation at the site level are summarized in Supplementary Tables S2 and S4.

At the implant level, PI was similar between study and control sides in β-TCP-treated (*P* = .240), and BCP-treated patients (*P = .*898; Table 3). PI was similar between study and control sides in bilaterally treated patients (*P* > .999; Table 3). KM was similar in study and control sides in β-TCP-treated (*P* > .999) and BCP-treated patients (*P* > .999; Table 3). KM was similar between study and control sides in bilaterally-treated patients (*P* = .218; Table 3).

#### Technical Complications

All initially placed suprastructures were still in function (~10 years). No implant, connection, or suprastructure-related technical complications were observed.

#### Graft and Residual Maxillary Sinus Characteristics

**Figure 2.**
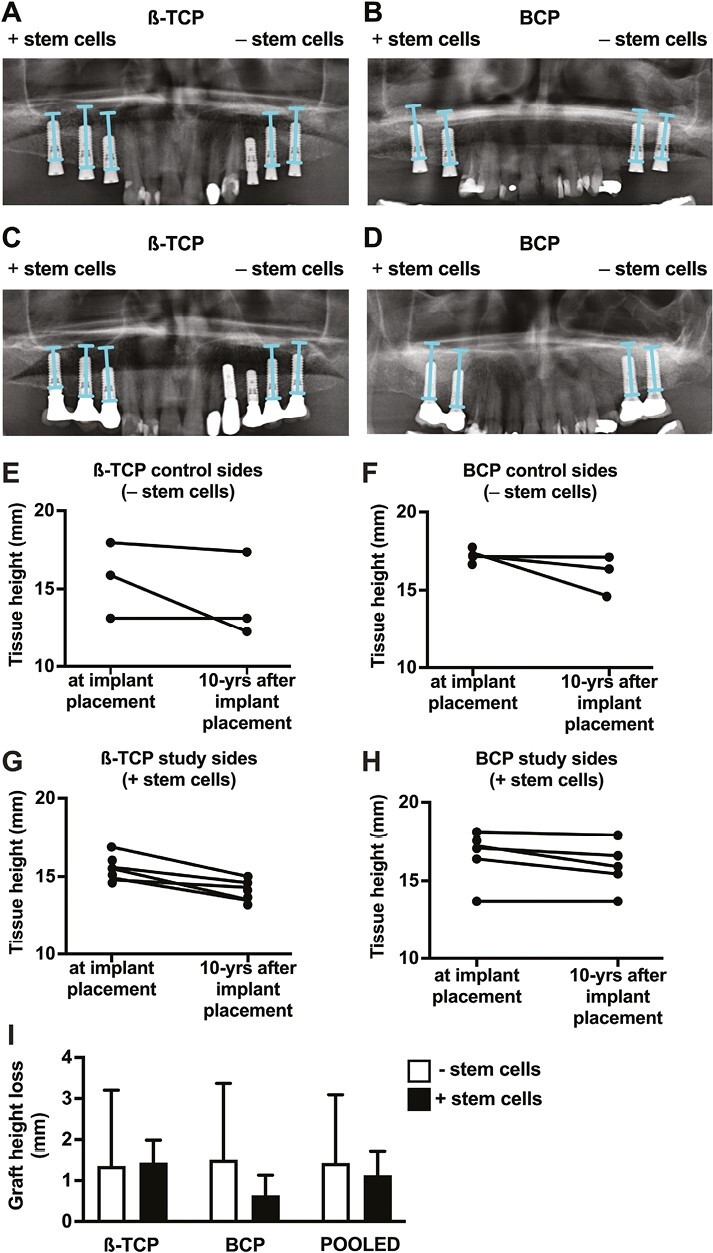
Tissue height, and graft height loss at the implant position at implant placement, and at 10 years after implant placement in β-TCP-treated and BCP-treated patients with and without stem cells. Representative panoramic radiographs: (**A**) Immediately after dental implant placement in a patient treated with β-TCP only and β-TCP plus stem cells. (**B**) Immediately after dental implant placement in a patient treated with BCP only and BCP plus stem cells. (**C**) 10-years after implant placement in a patient treated with β-TCP only and β-TCP plus stem cells. (**D**) 10-years after implant placement in a patient treated with BCP only and BCP plus stem cells. Blue line: Tissue height at implant position. Tissue height at implant placement, and 10-years after implant placement in patients-treated with (E) β-TCP only (*n* = 3), (F) BCP only (*n* = 3), (G) β-TCP plus stem cells (*n* = 5), (H) BCP plus stem cells (*n* = 5). (I) Graft height loss at 10-years after implant placement in β-TCP only (*n* = 3), β-TCP plus stem cells (*n* = 5), BCP only (*n* = 3), BCP plus stem cells (*n* = 5), pooled β-TCP and BCP only (bilaterally-treated; *n* = 6), pooled β-TCP and BCP plus stem cells (bilaterally-treated; *n* = 6). β-TCP, β-tricalcium phosphate; BCP, biphasic calcium phosphate; *n*, number of patients.

**Figure 3.**
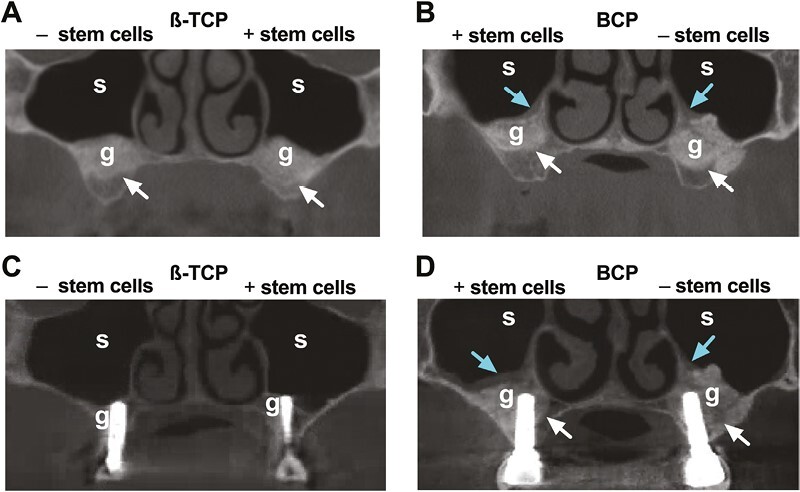
Cone beam computerized tomography (CBCT) scans of β-TCP-treated and BCP-treated patients with and without stem cells at 5 months post-MSFE and at 10-years after implant placement. CBCT scans of bilaterally treated patients with: (**A**) β-TCP only and β-TCP plus stem cells and (**B**) BCP plus stem cells and BCP only, at 5 months post-MSFE. In (A, B) homogenous radiopaque graft with a demarcation between residual native bone and the graft, and an air-filled residual maxillary sinus is visible. CBCT scans of bilaterally treated patients with: (**C**) β-TCP only and β-TCP plus stem cells, and (**D**) BCP plus stem cells and BCP only, at 10-years after implant placement. In (C, D) homogenous radiopaque graft, reduced graft volume, and an air-filled residual maxillary sinus is visible. MSFE, maxillary sinus floor elevation; β-TCP, β-tricalcium phosphate; BCP, biphasic calcium phosphate; g, graft; s, residual maxillary sinus; White arrows: demarcation original sinus floor; blue arrows: Schneiderian mucosal hypertrophy.

Schneiderian mucosal hypertrophy was observed at the 10-year follow-up in the study side of one β-TCP-treated patient (pt#4), and in study and control sides of 4 BCP-treated patients (pt#6: study and control sides; pt#7: control side; pt#8: study side; pt#10: study side; Fig. 2; Supplementary Table S4). Schneiderian mucosal hypertrophy was similar on radiographs pre-MSFE in all patients. No other pathologies were observed. Graft volume reduction was observed in all study and control sides in β-TCP-treated and BCP-treated patients at the 10-year follow-up, compared with 5 months post-MSFE (Fig. 3). Recognizable demarcation of the original maxillary sinus floor and homogenous graft structure was seen (Fig. 3A, B, D). Bone-like structured graft was observed in study and control sides in all β-TCP-treated patients (pt#1-pt#5), but only in study and control side in one out of 5 BCP-treated patients (pt#7; Fig. 3). A recognizable demarcation of radiopaque graft was observed in study sides in 3 β-TCP-treated patients (pt#3-pt#5), and in study and control sides in all BCP-treated patients (pt#6-pt#10; Fig. 3). No radiopacities or radiolucencies were seen in the graft material (Fig. 3). Radiologically air-filled maxillary sinus was observed in study and control sides in all patients. Qualitative radiological outcomes of SVF-supplementation are summarized in Supplementary Table S3.

#### Tissue Height and Graft Height Loss

Tissue height between dental implant placement and 10-year follow-up was reduced (reduction range: 5.2-14.1%) in study and control sides in all patients (Fig. 2A-H). Graft height loss between dental implant placement and 10-year follow-up was similar between study and control sides in β-TCP-treated (study: 1.35 ± 0.55 mm; control: 1.44 ± 1.88 mm; Fig. 2I), as well as in BCP-treated patients (study: 0.64 ± 0.49 mm; control: 1.51 ± 1.86 mm; Fig. 2J). Graft height loss between dental implant placement and 10-year follow-up was similar between study and control sides in bilaterally treated patients (study: 1.13 ± 0.58 mm; control: 1.43 ± 1.66 mm; Fig. 2K). Radiological outcomes of SVF-supplementation at the site level are summarized in Supplementary Table S2.

### Correlations Between Different Clinical and Radiological Outcomes

In control sides of β-TCP-treated patients, the following significant correlations were observed: SBI and PD (mm; *r = .*634), SBI and marginal bone loss (mm; *r = .*592), PD (mm) and marginal bone loss (mm; *r = .*738), KM (mm) and marginal bone loss (mm; *r* = −0.471; Table 4; *P* < .05). In study sides of β-TCP-treated patients, the only statistically significant correlation observed was between SBI and PD (mm; *r = .*525; Table 4; *P* < .05).

**Table 4.** Correlation coefficient matrix of measured clinical (plaque index, sulcus bleeding index, probing depth, width of keratinized mucosa) and radiological outcomes (marginal bone loss) of SVF-supplementation at 10-year follow-up.

		Control (- stem cells)				Study (+ stem cells)			
		Plaque index (PI)	Sulcus bleeding index (SBI)	Probing depth (PB; mm)	Width keratinized mucosa (KM; mm)	Plaque index (PI)	Sulcus bleeding index (SBI)	Probing depth (PD; mm)	Width keratinized mucosa (KM; mm)
β-TCP	Plaque index (PI)	—	—	—	—	—	—	—	—
	Sulcus bleeding index (SBI)	0.000	—	—	—	0.000	—	—	—
	Probing depth (PD; mm)	0.000	**0.634**	—	—	0.000	**0.525**	—	—
	Width keratinized mucosa (KM; mm)	0.000	— 0.278	— 0.012	—	0.000	— 0.165	— 0.107	—
	Marginal bone loss (mm)	0.000	**0.592**	**0.738**	**−0.471**	0.000	— 0.237	0.178	0.338
BCP	Plaque index (PI)	—	—	—	—	—	—	—	—
	Sulcus bleeding index (SBI)	0.000	—	—	—	0.000	—	—	—
	Probing depth (PD; mm)	0.000	**0.729**	—	—	0.000	**0.405**	—	—
	Width keratinized mucosa (KM; mm)	0.000	0.792	0.042	—	0.000	0.213	0.187	—
	Marginal bone loss (mm)	0.000	**0.607**	**0.853**	0.415	0.000	0.331	**0.526**	−0.081

Abbreviations: SVF, stromal vascular fraction; β-TCP, β-tricalcium phosphate; BCP, biphasic calcium phosphates. Bold values: significant at the .05 level.

In control sides of BCP-treated patients, the following significant correlations were observed: SBI and PD (mm; *r = .*729), SBI and marginal bone loss (mm; *r = .*607), PD (mm) and marginal bone loss (mm; *r = .*853; Table 4; *P* < .05). In study sides of BCP-treated patients, statistically significant correlation observed was observed between SBI and PD (mm; *r = .*405), PD (mm) and marginal bone loss (mm; *r = .*526; Table 4; *P* < .05).

There was no statistical difference in (baseline) alveolar bone height between β-TCP, β-TCP+SVF, BCP, and BCP+SVF-treated sides. Moreover, no correlation between baseline alveolar bone height and graft height loss was found.

## Discussion

In this 10-year prospective cohort study, SVF-supplementation in combination with calcium phosphate ceramics proofed to be safe for all patients undergoing MSFE. No adverse effects and pathology were found based on general health, clinical, and radiological assessments. A 100% implant survival rate was found in control sides of β-TCP-treated patients, and study and control sides of BCP-treated patients. A 92.9% implant survival rate (100% after 6 months follow-up) was found in study sides of β-TCP-treated patients, as a result of the loss of one implant. The failure was likely the result of premature loading by the temporary prosthesis.^7^ Since this implant was completely positioned in residual native bone, the cause of failure was unlikely related to SVF supplementation.

Stem cell-based therapies are associated with certain risks (eg, tumor, biodistribution, etc.).^17^ However, in our study no indications for safety concerns were found regarding SVF (containing ASCs)-application for MSFE. Similar clinical and radiological outcomes of dental implant success were observed in study and control sides of both β-TCP-treated and BCP-treated patients. Moreover, graft-related problems resulting from the application of autologous ASCs in cranioplasty, ie, graft resorption and late infection, have raised concerns about the long-term results of ASC-application in bone regeneration.^16^ Regardless of stem cell application, it should be noted that cranioplasty shows a 13-times higher complication rate requiring reoperation than MSFE using the lateral window technique due to the different nature of the surgical site, ie, large critical sized, tumorigenic defect.^16,25^ In our study, no patients reported any graft-related problems after MSFE, and showed 92.9-100% implant survival rate (100% following 6 months follow-up), which confirmed the successful long-term clinical outcomes after SVF supplementation in jawbone reconstruction.

This study investigated peri-implantitis, which is a biofilm-associated pathological condition occurring in the tissues around an osseointegrated implant, characterized by bleeding on probing and/or suppuration and progressive loss of supporting bone.^26^ We observed peri-implantitis in 3 out of 10 patients (30% prevalence), which agrees with prevalence rates (15-34%) reported by others.^27-29^ None of the established peri-implant disease risk factors (ie, poor oral hygiene, prosthesis overcontouring, history of periodontitis, diabetes, and smoking)^30,31^ were observed in these 3 peri-implantitis patients. One peri-implantitis patient did not receive implant maintenance for the last 2 years. Lack of implant maintenance may elevate the risk for peri-implantitis.^32^ Implant maintenance therapy of peri-implant mucositis may prevent peri-implantitis onset.^33^ Whether there is an association between keratinized mucosa and peri-implantitis is unclear.^32^ In our study, we did not find a correlation between inadequate width of keratinized mucosa (<2 mm) and increased marginal bone loss. There was no difference in peri-implantitis prevalence between study and control sides. Therefore, SVF-supplementation did not affect peri-implantitis prevalence at 10-year follow-up.

Our results showed no correlation between baseline alveolar bone height varying from 4 to 8 mm and implant survival or graft height loss at 10-year follow-up. Others have also shown that alveolar bone height of more than 4 mm does not affect implant survival after MSFE using the lateral window technique.^34^ Long-term graft height stability after MSFE is an important factor for implant success.^35^ We observed similar graft height loss in β-TCP (14%) and BCP-treated (12%) patients at 10-year follow-up. The graft height loss in ­β-TCP-treated patients was less compared to other reported studies at 5-year follow-up (28-39%).^36,37^ The difference in graft height loss could be related to the heterogeneity in our relatively small study population. The graft height loss in BCP-treated patients did agree with other reported studies (9-24%) using BCP with different HA/β-TCP ratio, ie, 20/80 (BCP20/80), 60/40 (BCP60/40), or 70/30 (BCP70/30), at 5-year^36^ or 6-year follow-up.^38^ A higher HA/β-TCP ratio seems to decrease graft height loss.^36,38^ SVF-supplementation seemed to inhibit (not significant) graft-height loss in study sides of BCP-treated patients but future studies with more patients are needed for verification.

We observed Schneiderian mucosal hypertrophy (>1 mm) pre-operatively in 5 patients (prevalence rate: 50%), which is in line with a prevalence rate (55%) reported by others.^39^ The extent of pre-existing Schneiderian mucosal hypertrophy did not increase after MSFE during 10-year ­follow-up in all patients. Schneiderian membrane hypertrophy is characteristic of maxillary sinusitis, but also common in ­asymptomatic patients.^39^ Moreover, no abnormalities (ie, mucosal cysts, polyps, bone lesions, neoplasms, and antroliths) in the maxillary sinus were observed after MSFE with SVF supplementation. This indicates that SVF supplementation did not induce abnormalities in the maxillary sinus, suggesting that no pathologic condition had developed and that SVF-supplementation can be safely used in MSFE.

An expected moderate-to-strong correlation (*r = .*5-1) between sulcus bleeding and marginal bone loss was found at control sides, but not study sides, of β-TCP-treated patients. The absence of this correlation in the study sides might suggest a positive effect of SVF-supplementation on the peri-implant condition, since sulcus bleeding and marginal bone loss are symptoms of peri-implant tissue inflammation. Future studies with more patients are needed to reveal a possible effect of SVF-supplementation on peri-implant condition.

The study design resulted in potential observer bias, which is a limitation. The surgeons were not blinded for the type of graft during MSFE, and had prior knowledge of the research aims. Data collection and analysis at 10-year follow-up were performed blinded, thereby excluding observer bias. A limitation of our study was that no baseline radiographic and probing data after the first year of implant loading were recorded. Therefore, onset and progression of peri-implant disease could not be determined.

Our study used enzymatic preparation of ASCs, which is still the most frequently used method to isolate ASCs from adipose tissue.^40^ However, in most countries this method is considered as “more than minimal manipulation” of stem cells.^40^ The extensive use and manipulation of stem cells within a clinical setting has been hindered by the GMP regulations regarding “cell manufacturing.” These regulations are not applicable to “minimally manipulated” stem cells according to the European Parliament and Council (EC regulation no. 1394/2007). Enzymatic preparation of ASCs, therefore, falls within the definition of an advanced therapy medicinal product according to the European Parliament and Council. Enzymatic preparation of ASC was considered a limitation of our study, based on European legislation. As an alternative method, mechanical disaggregation of the adipose tissue into small fat particles, so-called micro-fragmented fat (MFAT), has been investigated to decrease regulatory burden, and shorten the translation into the clinical setting.^41-43^ Intact microarchitecture of MFAT preserves a similar or even higher number of regenerative cells than the enzymatically derived SVF.^44-46^

Our previous phase I study used general anesthesia to avoid complications during adipose tissue procurement and MSFE surgery.^7^ Clinical studies using local anesthesia for liposuction are currently being undertaken.^47,48^ This may broaden the applicability of SVF-supplementation to calcium phosphate ceramics by decreasing the treatment burden for patients, and improving the cost-effectiveness of the treatment.

Although the potential of ASCs evokes high expectations for the application of cellular bone tissue engineering,^8^ clinical evidence of abdominal derived-ASC-application for oral and maxillofacial bone regeneration is limited to a few successful short-term outcomes (≤3-year follow-up).^7,13,49^ To the best of our knowledge, no long-term results have been reported. Stem cell application in regenerative medicine has raised safety concerns, eg, tumorigenic potential and biodistribution.^17^ This can only manifest itself in the long term. Therefore, clinical studies investigating long-term safety and efficacy are crucial before continuing with clinical application of (adipose) stem cells.

The primary objectives of the previous phase I clinical study were feasibility and safety of SVF in combination with calcium phosphate ceramics in MSFE.^7^ A power analysis could not be carried out, since there was no specific parameter to compare the groups. Also, a direct comparison between the 4 graft types, ie, β-TCP, β-TCP+SVF, BCP, and BCP+SVF, was not done earlier, and therefore a multi-parameter evaluation was performed to identify potential differences in treatment outcome in an unbiased manner. A sample size of 10 patients is commonly accepted for a (pilot) phase I clinical study. Regardless of this low number of patients, the encouraging finding that SVF supplementation is safe in both short- and long-term certainly warrants further studies to evaluate whether eg, using higher dosages of SVF, altered scaffold properties, or application of other adipose tissue processing methods may enhance bone formation without increasing side effects.

## Conclusion

This study demonstrated for the first time the long-term safety of SVF-supplementation in combination with calcium phosphate ceramics in MSFE using the lateral window technique for jawbone reconstruction. SVF-supplementation enhanced bone regeneration in the short term, as shown in our previous study,^7^ and led to no abnormalities, clinically and radiologically, in the long term. Future studies with more patients and higher SVF dosages might further improve efficacy and open new possibilities for a variety of cell-based bone tissue engineering applications.

## Supplementary Material

szad045_suppl_Supplementary_Table_S1

szad045_suppl_Supplementary_Table_S2

szad045_suppl_Supplementary_Table_S3

szad045_suppl_Supplementary_Table_S4

szad045_suppl_Supplementary_Table_S5

## Data Availability

The data that support the findings of this study are available on request from the corresponding author. The data are not publicly available due to privacy or ethical restrictions.
