# Supplementary material for: Long-Term Safety of Bone Regeneration Using Autologous Stromal Vascular Fraction and Calcium Phosphate Ceramics: A 10-Year Prospective Cohort Study
Source: Stem Cells Transl Med. 2023 Aug 1;12(9):617–30. doi: 10.1093/stcltm/szad045 (PMC10502529; doi:10.1093/stcltm/szad045)
Supplement: szad045_suppl_Supplementary_Table_S1 [file szad045_suppl_supplementary_table_s1.docx]

**Table S1** Radiological data per patient

|  | Panoramic radiograph | | | | | | | |  | CBCT-scan | | | | |
| --- | --- | --- | --- | --- | --- | --- | --- | --- | --- | --- | --- | --- | --- | --- |
| Pt# | T=0 | T=1 | T=2 | T=3 | T=4 | T=5 | T=6 | T=7 |  | T=0 | T=2 | T=5 | T=6 | T=7 |
| 1 | x | x | x | x | x | x |  | x |  | x | x |  |  | x |
| 2 | x | x | x | x | x | x | x | x |  | x | x |  | x | x |
| 3 | x | x | x | x | x |  | x | x |  | x | x | x | x | x |
| 4 | x | x | x | x | x | x | x | x |  | x | x |  |  | x |
| 5 | x | x | x | x | x | x |  | x |  | x | x | x |  | x |
| 6 | x | x | x | x | x | x | x | x |  | x | x | x | x | x |
| 7 | x | x | x | x | x |  |  | x |  | x | x |  |  | x |
| 8 | x | x | x | x | x |  |  | x |  | x | x |  |  | x |
| 9 | x | x | x | x | x |  |  | x |  | x | x |  |  | x |
| 10 | x | x | x | x | x | x | x | x |  | x | x |  | x | x |

Panoramic radiograph and cone beam-computerized tomography (CBCT)-scan taken per patient per visit. T0=intake; T1=directly after maxillary sinus floor elevation (MSFE); T2=5-months post-MSFE (ridgemapping visit); T3=6-months post-MSFE(directly after implant placement); T4=9-months post-MSFE(osseointegration check visit); T5=18-months post-MSFE (1-year after implant placement); T6=36-months post-MSFE (2.5-years after implant placement); T7=126-months post-MSFE (10-years after implant placement).
