# Supplementary material for: Long-Term Safety of Bone Regeneration Using Autologous Stromal Vascular Fraction and Calcium Phosphate Ceramics: A 10-Year Prospective Cohort Study
Source: Stem Cells Transl Med. 2023 Aug 1;12(9):617–30. doi: 10.1093/stcltm/szad045 (PMC10502529; doi:10.1093/stcltm/szad045)
Supplement: szad045_suppl_Supplementary_Table_S2 [file szad045_suppl_supplementary_table_s2.docx]

**Table S2.** Radiological outcomes of SVF-supplementation at the site level at 10-years follow-up

|  |  | Marginal bone loss (mm) | | | | | | |  | Tissue height (mm) at implant position | | | | |  | Graft height loss (mm) at implant position | | | | |  | Tissue height (mm) at inter-implant (distal) position | | | | |  | Graft height loss (mm) at inter-implant (distal) position | | | | |
| --- | --- | --- | --- | --- | --- | --- | --- | --- | --- | --- | --- | --- | --- | --- | --- | --- | --- | --- | --- | --- | --- | --- | --- | --- | --- | --- | --- | --- | --- | --- | --- | --- |
|  |  | Control | | |  | Study | | |  | Control | |  | Study | |  | Control | |  | Study | |  | Control | |  | Study | |  | Control | |  | Study | |
|  |  | (- stem cells) | | |  | (+ stem cells) | | |  | (- stem cells) | |  | (+ stem cells) | |  | (- stem cells) | |  | (+ stem cells) | |  | (- stem cells) | |  | (+ stem cells) | |  | (- stem cells) | |  | (+ stem cells) | |
| Pt# | Graft | Imp | M | D |  | Imp | M | D |  | Imp |  |  | Imp |  |  | Imp |  |  | Imp |  |  | Imp |  |  | Imp |  |  | Imp |  |  | Imp |  |
| 1 | ß-TCP | 14 | 3 | 3 |  | 24 | 0 | 0 |  | 14 | n.a. |  | 24 | n.a. |  | 14 | n.a. |  | 24 | n.a. |  | 14 | 13 |  | 24 | 17 |  | 14 | 2 |  | 24 | 0 |
|  | ß-TCP | 15 | 4 | 4 |  | 25 | 1 | 2 |  | 15 | 12 |  | 25 | 14 |  | 15 | 4 |  | 25 | 2 |  | 15 | 14 |  | 25 | 13 |  | 15 | 2 |  | 25 | 1 |
|  | ß-TCP | 16 | 3 | 4 |  | 26 | 1 | 0 |  | 16 | 12 |  | 26 | 13 |  | 16 | 4 |  | 26 | 1 |  | 16 | 11 |  | 26 | 10 |  | 16 | 7 |  | 26 | 2 |
|  |  |  |  |  |  |  |  |  |  |  |  |  |  |  |  |  |  |  |  |  |  |  |  |  |  |  |  |  |  |  |  |  |
| 2 | ß-TCP | 24 | 2 | 1 |  | 14 | 0 | 1 |  | 24 | n.a. |  | 14 | 16 |  | 24 | n.a. |  | 14 | 1 |  | 24 | 16 |  | 14 | 15 |  | 24 | 3 |  | 14 | 2 |
|  | ß-TCP | 25 | 1 | 0 |  | 15 | 0 | 0 |  | 25 | 18 |  | 15 | 16 |  | 25 | 0 |  | 15 | 2 |  | 25 | 16 |  | 15 | 14 |  | 25 | 0 |  | 15 | 4 |
|  | ß-TCP | 26 | 0 | 0 |  | 16 | 1 | 1 |  | 26 | 17 |  | 16 | 13 |  | 26 | 1 |  | 16 | 4 |  | 26 | 15 |  | 16 | 13 |  | 26 | 1 |  | 16 | 1 |
|  |  |  |  |  |  |  |  |  |  |  |  |  |  |  |  |  |  |  |  |  |  |  |  |  |  |  |  |  |  |  |  |  |
| 3 | ß-TCP | 14 | 1 | 3 |  | 25 | 0 | 1 |  | 14 | n.a. |  | 25 | 16 |  | 14 | n.a. |  | 25 | 0 |  | 14 | 15 |  | 25 | 17 |  | 14 | 2 |  | 25 | 0 |
|  | ß-TCP | 15 | 1 | 1 |  | 26 | 1 | 1 |  | 15 | 14 |  | 26 | 14 |  | 15 | 0 |  | 26 | 2 |  | 15 | 12 |  | 26 | 17 |  | 15 | 0 |  | 26 | 2 |
|  | ß-TCP | 16 | 0 | 0 |  | 27 | 1 | 1 |  | 16 | 12 |  | 27 | 13 |  | 16 | 0 |  | 27 | 0 |  | 16 | 12 |  | 27 | 16 |  | 16 | 3 |  | 27 | 1 |
|  |  |  |  |  |  |  |  |  |  |  |  |  |  |  |  |  |  |  |  |  |  |  |  |  |  |  |  |  |  |  |  |  |
| 4 | ß-TCP |  |  |  |  | 24 | 3 | 3 |  |  |  |  | 24 | n.a. |  |  |  |  | 24 | n.a. |  |  |  |  | 24 | 20 |  |  |  |  | 24 | 0 |
|  | ß-TCP |  |  |  |  | 25 | 0 | 1 |  |  |  |  | 25 | 17 |  |  |  |  | 25 | 2 |  |  |  |  | 25 | 13 |  |  |  |  | 25 | 2 |
|  | ß-TCP |  |  |  |  | 26 | 0 | 0 |  |  |  |  | 26 | 12 |  |  |  |  | 26 | 0 |  |  |  |  | 26 | 9 |  |  |  |  | 26 | 0 |
|  |  |  |  |  |  |  |  |  |  |  |  |  |  |  |  |  |  |  |  |  |  |  |  |  |  |  |  |  |  |  |  |  |
| 5 | ß-TCP |  |  |  |  | 15 | 0 | 3 |  |  |  |  | 15 | 15 |  |  |  |  | 15 | 2 |  |  |  |  | 15 | 13 |  |  |  |  | 15 | 1 |
|  | ß-TCP |  |  |  |  | 16 | 0 | 0 |  |  |  |  | 16 | 12 |  |  |  |  | 16 | 2 |  |  |  |  | 16 | 14 |  |  |  |  | 16 | 0 |
|  |  |  |  |  |  |  |  |  |  |  |  |  |  |  |  |  |  |  |  |  |  |  |  |  |  |  |  |  |  |  |  |  |
| 6 | BCP | 24 | 0 | 0 |  | 14 | 0 | 0 |  | 24 | 17 |  | 14 | n.a. |  | 24 | 0 |  | 14 | n.a. |  | 24 | 18 |  | 14 | 16 |  | 24 | 1 |  | 14 | 0 |
|  | BCP | 26 | 0 | 0 |  | 15 | 0 | 0 |  | 26 | 17 |  | 15 | 16 |  | 26 | 0 |  | 15 | 1 |  | 26 | 17 |  | 15 | 13 |  | 26 | 0 |  | 15 | 2 |
|  | BCP |  |  |  |  | 16 | 0 | 0 |  |  |  |  | 16 | 15 |  |  |  |  | 16 | 1 |  |  |  |  | 16 | 13 |  |  |  |  | 16 | 2 |
|  |  |  |  |  |  |  |  |  |  |  |  |  |  |  |  |  |  |  |  |  |  |  |  |  |  |  |  |  |  |  |  |  |
| 7 | BCP | 25 | 0 | 0 |  | 15 | 0 | 0 |  | 25 | 16 |  | 15 | n.a. |  | 25 | 1 |  | 15 | n.a. |  | 25 | 15 |  | 15 | 15 |  | 25 | 1 |  | 15 | 0 |
|  | BCP | 26 | 0 | 0 |  | 16 | 0 | 2 |  | 26 | 15 |  | 16 | 16 |  | 26 | 1 |  | 16 | 1 |  | 26 | 17 |  | 16 | 16 |  | 26 | 0 |  | 16 | 1 |
|  | BCP | 27 | 5 | 6 |  | 17 | 3 | 2 |  | 27 | 13 |  | 17 | 16 |  | 27 | 6 |  | 17 | 1 |  | 27 | 14 |  | 17 | 15 |  | 27 | 6 |  | 17 | 1 |
|  |  |  |  |  |  |  |  |  |  |  |  |  |  |  |  |  |  |  |  |  |  |  |  |  |  |  |  |  |  |  |  |  |
| 8 | BCP |  |  |  |  | 14 | 0 | 0 |  |  |  |  | 14 | 18 |  |  |  |  | 14 | 0 |  |  |  |  | 14 | 17 |  |  |  |  | 14 | 1 |
|  | BCP |  |  |  |  | 15 | 0 | 0 |  |  |  |  | 15 | 16 |  |  |  |  | 15 | 1 |  |  |  |  | 15 | 16 |  |  |  |  | 15 | 0 |
|  | BCP |  |  |  |  | 16 | 0 | 0 |  |  |  |  | 16 | 16 |  |  |  |  | 16 | 0 |  |  |  |  | 16 | 17 |  |  |  |  | 16 | 0 |
|  |  |  |  |  |  |  |  |  |  |  |  |  |  |  |  |  |  |  |  |  |  |  |  |  |  |  |  |  |  |  |  |  |
| 9 | BCP |  |  |  |  | 23 | 1 | 1 |  |  |  |  | 23 | n.a. |  |  |  |  | 23 | n.a. |  |  |  |  | 23 | n.a. |  |  |  |  | 23 | n.a. |
|  | BCP |  |  |  |  | 25 | 1 | 1 |  |  |  |  | 25 | 14 |  |  |  |  | 25 | 0 |  |  |  |  | 25 | 14 |  |  |  |  | 25 | 1 |
|  | BCP |  |  |  |  | 26 | 1 | 1 |  |  |  |  | 26 | 13 |  |  |  |  | 26 | 0 |  |  |  |  | 26 | 12 |  |  |  |  | 26 | 1 |
|  |  |  |  |  |  |  |  |  |  |  |  |  |  |  |  |  |  |  |  |  |  |  |  |  |  |  |  |  |  |  |  |  |
| 10 | BCP | 25 | 0 | 0 |  | 15 | 0 | 0 |  | 25 | 22 |  | 15 | 18 |  | 25 | 0 |  | 15 | 0 |  | 25 | 17 |  | 15 | 18 |  | 25 | 0 |  | 15 | 0 |
|  | BCP | 26 | 0 | 0 |  | 16 | 0 | 0 |  | 26 | 19 |  | 16 | 18 |  | 26 | 2 |  | 16 | 0 |  | 26 | 17 |  | 16 | 18 |  | 26 | 1 |  | 16 | 0 |

For each implant the marginal bone loss (mm) was determined at the mesial and distal implant surface, and the tissue height (mm) and graft height loss (mm) at the implant mid-axis and at inter-implant (distal) position on the panoramic radiograph. SVF, stromal vascular fraction, Pt#, patient number; Imp, implant position; M, mesial; D, distal; ß-TCP, ß-tricalcium phosphate; BCP, biphasic calcium phosphates.
