# Supplementary material for: Long-Term Safety of Bone Regeneration Using Autologous Stromal Vascular Fraction and Calcium Phosphate Ceramics: A 10-Year Prospective Cohort Study
Source: Stem Cells Transl Med. 2023 Aug 1;12(9):617–30. doi: 10.1093/stcltm/szad045 (PMC10502529; doi:10.1093/stcltm/szad045)
Supplement: szad045_suppl_Supplementary_Table_S3 [file szad045_suppl_supplementary_table_s3.docx]

**Table S3** Qualitative radiological outcomes of SVF-supplementation at 10-years follow-up on panoramic radiograph and cone beam computerized tomography (CBCT)-scan.

|  |  |  | Pt#1 | | | | |  | Pt#2 | | | | | |  | Pt#3 | | | | | |  | Pt#4 | |  | Pt#5 | |
| --- | --- | --- | --- | --- | --- | --- | --- | --- | --- | --- | --- | --- | --- | --- | --- | --- | --- | --- | --- | --- | --- | --- | --- | --- | --- | --- | --- |
|  |  |  | Control | |  | Study | |  | Control | |  | Study | | |  | Control | |  | Study | | |  | Study | |  | Study | |
|  |  |  | - stem cells | |  | + stem cells | |  | - stem cells | |  | + stem cells | | |  | - stem cells | |  | + stem cells | | |  | + stem cells | |  | + stem cells | |
|  |  |  | Imp | |  | Imp | |  | Imp | |  | Imp | | |  | Imp | |  | Imp | | |  | Imp | |  | Imp | |
|  |  |  | 15 | 16 |  | 25 | 26 |  | 25 | 26 |  | 14 | 15 | 16 |  | 15 | 16 |  | 25 | 26 | 27 |  | 25 | 26 |  | 15 | 16 |
| ß-TCP | Panoramic radiograph | |  |  |  |  |  |  |  |  |  |  |  |  |  |  |  |  |  |  |  |  |  |  |  |  |  |
|  |  | Irregularities sinus floor |  |  |  |  |  |  |  |  |  |  |  |  |  |  |  |  |  |  |  |  |  |  |  |  |  |
|  |  | Recognizable demarcation original sinusfloor | x | x |  | x | x |  | x | x |  | x | x | x |  | x | x |  | x | x | x |  | x | x |  | x | x |
|  |  | Homogenous graft structure | x | x |  | x | x |  | x | x |  | x | x | x |  | x | x |  | x | x | x |  | x | x |  | x | x |
|  |  | Recognizable demarcation radio-opaque graft |  |  |  |  |  |  |  |  |  |  |  |  |  |  |  |  |  |  |  |  | x | x |  | x | x |
|  |  | Bone-like structured graft | x | x |  | x | x |  | x | x |  | x | x | x |  | x | x |  | x | x | x |  |  |  |  |  |  |
|  |  | Radiopacities in the graft |  |  |  |  |  |  |  |  |  |  |  |  |  |  |  |  |  |  |  |  |  |  |  |  |  |
|  |  | Radiolucencies in the graft |  |  |  |  |  |  |  |  |  |  |  |  |  |  |  |  |  |  |  |  |  |  |  |  |  |
|  |  | Scalloping of the graft |  |  |  |  |  |  |  |  |  |  |  |  |  |  |  |  | x | x | x |  |  | x |  |  |  |
|  |  | Air-filled residual maxillary sinus | x | x |  | x | x |  | x | x |  | x | x | x |  | x | x |  | x | x | x |  | x | x |  | x | x |
|  |  | Schneiderian mucosal hypertrophy |  |  |  |  |  |  |  |  |  |  |  |  |  |  |  |  |  |  |  |  | x | x |  |  |  |
|  |  | Pathology |  |  |  |  |  |  |  |  |  |  |  |  |  |  |  |  |  |  |  |  |  |  |  |  |  |
|  |  |  |  |  |  |  |  |  |  |  |  |  |  |  |  |  |  |  |  |  |  |  |  |  |  |  |  |
|  | CBCT-scan | |  |  |  |  |  |  |  |  |  |  |  |  |  |  |  |  |  |  |  |  |  |  |  |  |  |
|  |  | Irregularities sinus floor |  |  |  |  |  |  |  |  |  |  |  |  |  |  |  |  |  |  |  |  |  |  |  |  |  |
|  |  | Recognizable demarcation original trapdoor |  |  |  |  |  |  |  |  |  |  | x |  |  |  | x |  |  |  | x |  |  |  |  |  |  |
|  |  | Recognizable demarcation original sinusfloor |  |  |  |  |  |  |  |  |  |  |  |  |  |  |  |  |  |  |  |  |  |  |  |  | x |
|  |  | Homogenous graft structure | x | x |  | x | x |  | x | x |  | x | x | x |  | x | x |  | x | x |  |  | x | x |  | x | x |
|  |  | Recognizable demarcation radio-opaque graft |  |  |  |  |  |  |  |  |  |  |  |  |  |  |  |  |  |  | x |  |  |  |  |  |  |
|  |  | Bone-like structured graft | x | x |  | x | x |  | x | x |  | x | x | x |  | x | x |  | x | x |  |  | x | x |  | x | x |
|  |  | Radiopacities in the graft |  |  |  |  |  |  |  |  |  |  |  |  |  |  |  |  |  |  |  |  |  |  |  |  |  |
|  |  | Radiolucencies in the graft |  |  |  |  |  |  |  |  |  |  |  |  |  |  |  |  |  |  |  |  |  |  |  |  |  |
|  |  | Scalloping of the graft (buc-pal) |  | x |  |  | x |  |  |  |  |  |  |  |  |  |  |  |  |  |  |  |  | x |  |  |  |
|  |  | Air-filled residual maxillary sinus | x | x |  | x | x |  | x | x |  | x | x | x |  | x | x |  | x | x | x |  | x | x |  | x | x |
|  |  | Schneiderian mucosal hypertrophy |  |  |  |  |  |  |  |  |  |  |  |  |  |  |  |  |  |  |  |  | x | x |  |  |  |
|  |  | Pathology |  |  |  |  |  |  |  |  |  |  |  |  |  |  |  |  |  |  |  |  |  |  |  |  |  |
|  |  |  |  |  |  |  |  |  |  |  |  |  |  |  |  |  |  |  |  |  |  |  |  |  |  |  |  |
|  |  |  | Pt#6 | | | | |  | Pt#7 | | | | | |  | Pt#8 | | |  | Pt#9 | |  | Pt#10 | | | | |
|  |  |  | Control | |  | Study | |  | Control | | |  | Study | |  | Study | | |  | Study | |  | Control | |  | Study | |
|  |  |  | - stem cells | |  | + stem cells | |  | - stem cells | | |  | + stem cells | |  | + stem cells | | |  | + stem cells | |  | - stem cells | |  | + stem cells | |
|  |  |  | Imp | |  | Imp | |  | Imp | |  |  | Imp | |  | Imp | | |  | Imp | |  | Imp | |  | Imp | |
|  |  |  | 24 | 26 |  | 15 | 16 |  | 25 | 26 |  |  | 16 | 17 |  | 14 | 15 | 16 |  | 25 | 26 |  | 25 | 26 |  | 14 | 15 |
| BCP | Panoramic radiograph | |  |  |  |  |  |  |  |  |  |  |  |  |  |  |  |  |  |  |  |  |  |  |  |  |  |
|  |  | Irregularities sinus floor |  |  |  |  |  |  |  |  |  |  |  |  |  |  |  |  |  | x | x |  | x | x |  |  |  |
|  |  | Recognizable demarcation original sinusfloor | x | x |  | x | x |  | x | x | x |  | x | x |  | x | x | x |  | x | x |  | x | x |  | x | x |
|  |  | Homogenous graft structure | x | x |  | x | x |  | x | x | x |  | x | x |  | x | x | x |  | x | x |  | x | x |  | x | x |
|  |  | Recognizable demarcation radio-opaque graft | x | x |  | x | x |  | x | x | x |  | x | x |  | x | x | x |  | x | x |  | x | x |  | x | x |
|  |  | Bone-like structured graft |  |  |  |  |  |  |  |  |  |  |  |  |  |  |  |  |  |  |  |  |  |  |  |  |  |
|  |  | Radiopacities in the graft |  |  |  |  |  |  |  |  |  |  |  |  |  |  |  |  |  |  |  |  |  |  |  |  |  |
|  |  | Radiolucencies in the graft |  |  |  |  |  |  |  |  |  |  |  |  |  |  |  |  |  |  |  |  |  |  |  |  |  |
|  |  | Scalloping of the graft |  |  |  |  |  |  |  |  |  |  |  |  |  |  |  |  |  |  |  |  |  |  |  |  |  |
|  |  | Air-filled residual maxillary sinus | x | x |  | x | x |  | x | x | x |  | x | x |  | x | x | x |  | x | x |  | x | x |  | x | x |
|  |  | Schneiderian mucosal hypertrophy | x | x |  | x | x |  |  |  |  |  |  |  |  | x | x | x |  |  |  |  |  |  |  |  |  |
|  |  | Pathology |  |  |  |  |  |  |  |  |  |  |  |  |  |  |  |  |  |  |  |  |  |  |  |  |  |
|  |  |  |  |  |  |  |  |  |  |  |  |  |  |  |  |  |  |  |  |  |  |  |  |  |  |  |  |
|  | CBCT-scan | |  |  |  |  |  |  |  |  |  |  |  |  |  |  |  |  |  |  |  |  |  |  |  |  |  |
|  |  | Irregularities sinus floor |  |  |  |  |  |  |  |  |  |  |  |  |  |  |  |  |  | x | x |  |  | x |  |  |  |
|  |  | Recognizable demarcation original trapdoor | x | x |  | x | x |  |  |  |  |  |  |  |  |  |  |  |  |  |  |  |  |  |  |  |  |
|  |  | Recognizable demarcation original sinusfloor |  |  |  |  |  |  |  |  |  |  |  |  |  | x | x |  |  | x | x |  | x | x |  | x | x |
|  |  | Homogenous graft structure | x | x |  | x |  |  | x | x | x |  | x | x |  | x | x | x |  |  |  |  |  |  |  | x | x |
|  |  | Recognizable demarcation radiopaque graft | x | x |  | x | x |  |  |  |  |  |  |  |  |  |  |  |  |  |  |  | x | x |  | x | x |
|  |  | Bone-like structured graft |  |  |  |  |  |  | x | x | x |  | x | x |  |  |  |  |  |  |  |  |  |  |  |  |  |
|  |  | Radiopacities in the graft |  |  |  |  |  |  |  |  |  |  |  |  |  |  |  |  |  |  |  |  |  |  |  |  |  |
|  |  | Radiolucencies in the graft |  |  |  |  |  |  |  |  |  |  |  |  |  |  |  |  |  |  |  |  |  |  |  |  |  |
|  |  | Scalloping of the graft (buc-pal) |  |  |  |  |  |  | x | x | x |  | x | x |  |  | x | x |  |  |  |  |  |  |  |  |  |
|  |  | Air-filled residual maxillary sinus | x | x |  | x | x |  | x | x | x |  | x | x |  | x | x | x |  | x | x |  | x | x |  | x | x |
|  |  | Schneiderian mucosal hypertrophy | x | x |  | x | x |  | x | x | x |  |  |  |  | x |  |  |  |  |  |  | x |  |  |  |  |
|  |  | Pathology |  |  |  |  |  |  |  |  |  |  |  |  |  |  |  |  |  |  |  |  |  |  |  |  |  |

SVF, stromal vascular fraction; β-TCP, ß-tricalcium phosphate; BCP, biphasic calcium phosphate; CBCT, cone beam computerized tomography.
