# Supplementary material for: Long-Term Safety of Bone Regeneration Using Autologous Stromal Vascular Fraction and Calcium Phosphate Ceramics: A 10-Year Prospective Cohort Study
Source: Stem Cells Transl Med. 2023 Aug 1;12(9):617–30. doi: 10.1093/stcltm/szad045 (PMC10502529; doi:10.1093/stcltm/szad045)
Supplement: szad045_suppl_Supplementary_Table_S4 [file szad045_suppl_supplementary_table_s4.docx]

**Table S4** Clinical outcomes of SVF-supplementation at the site level at 10-years follow-up

|  |  |  | Plaque Index | | | | | | | | | |  |  | Probing Depth (mm) | | | | | | | | | |  |  | Sulcus Bleeding Index | | | | | | | | | |
| --- | --- | --- | --- | --- | --- | --- | --- | --- | --- | --- | --- | --- | --- | --- | --- | --- | --- | --- | --- | --- | --- | --- | --- | --- | --- | --- | --- | --- | --- | --- | --- | --- | --- | --- | --- | --- |
|  |  |  | Control | | | |  |  | Study | | | |  |  | Control | | | |  |  | Study | | | |  |  | Control | | | |  |  | Study | | | |
|  |  |  | (– stem cells) | | | |  |  | (+ stem cells) | | | |  |  | (– stem cells) | | | |  |  | (+ stem cells) | | | |  |  | (– stem cells) | | | |  |  | (+ stem cells) | | | |
| Pt# | Graft | Imp | M | B | D | P |  | Imp | M | B | D | P |  | Imp | M | B | D | P |  | Imp | M | B | D | P |  | Imp | M | B | D | P |  | Imp | M | B | D | P |
| 1 | ß-TCP | 14 | 0 | 0 | 0 | 0 |  | 24 | 0 | 0 | 0 | 0 |  | 14 | 7 | 5 | 7 | 4 |  | 24 | 5 | 3 | 3 | 3 |  | 14 | 1 | 2 | 2 | 1 |  | 24 | 1 | 1 | 0 | 1 |
|  | ß-TCP | 15 | 0 | 0 | 0 | 0 |  | 25 | 0 | 0 | 0 | 0 |  | 15 | 4 | 6 | 8 | 6 |  | 25 | 5 | 3 | 4 | 4 |  | 15 | 2 | 2 | 2 | 2 |  | 25 | 1 | 1 | 1 | 1 |
|  | ß-TCP | 16 | 0 | 0 | 0 | 0 |  | 26 | 0 | 0 | 0 | 0 |  | 16 | 7 | 4 | 7 | 6 |  | 26 | 5 | 3 | 5 | 4 |  | 16 | 2 | 2 | 2 | 2 |  | 26 | 2 | 1 | 1 | 2 |
|  |  |  |  |  |  |  |  |  |  |  |  |  |  |  |  |  |  |  |  |  |  |  |  |  |  |  |  |  |  |  |  |  |  |  |  |  |
| 2 | ß-TCP | 24 | 0 | 0 | 0 | 0 |  | 14 | 0 | 0 | 0 | 0 |  | 24 | 6 | 4 | 5 | 5 |  | 14 | 2 | 2 | 4 | 2 |  | 24 | 2 | 2 | 2 | 2 |  | 14 | 0 | 0 | 1 | 0 |
|  | ß-TCP | 25 | 0 | 0 | 0 | 0 |  | 15 | 0 | 0 | 0 | 0 |  | 25 | 5 | 4 | 5 | 5 |  | 15 | 2 | 2 | 4 | 2 |  | 25 | 1 | 0 | 0 | 2 |  | 15 | 0 | 0 | 1 | 0 |
|  | ß-TCP | 26 | 0 | 0 | 0 | 0 |  | 16 | 0 | 0 | 0 | 0 |  | 26 | 3 | 3 | 3 | 4 |  | 16 | 2 | 3 | 2 | 3 |  | 26 | 2 | 0 | 0 | 2 |  | 16 | 0 | 1 | 0 | 0 |
|  |  |  |  |  |  |  |  |  |  |  |  |  |  |  |  |  |  |  |  |  |  |  |  |  |  |  |  |  |  |  |  |  |  |  |  |  |
| 3 | ß-TCP | 14 | 0 | 0 | 0 | 0 |  | 25 | 0 | 0 | 0 | 0 |  | 14 | 5 | 2 | 5 | 3 |  | 25 | 7 | 2 | 4 | 2 |  | 14 | 1 | 0 | 0 | 0 |  | 25 | 1 | 0 | 0 | 0 |
|  | ß-TCP | 15 | 0 | 0 | 0 | 0 |  | 26 | 0 | 0 | 0 | 0 |  | 15 | 4 | 2 | 4 | 2 |  | 26 | 4 | 3 | 4 | 4 |  | 15 | 0 | 0 | 0 | 0 |  | 26 | 1 | 0 | 1 | 1 |
|  | ß-TCP | 16 | 0 | 0 | 0 | 0 |  | 27 | 0 | 0 | 0 | 0 |  | 16 | 4 | 2 | 4 | 2 |  | 27 | 4 | 2 | 3 | 5 |  | 16 | 0 | 0 | 0 | 0 |  | 27 | 1 | 0 | 0 | 1 |
|  |  |  |  |  |  |  |  |  |  |  |  |  |  |  |  |  |  |  |  |  |  |  |  |  |  |  |  |  |  |  |  |  |  |  |  |  |
| 4 | ß-TCP |  |  |  |  |  |  | 24 | 0 | 0 | 0 | 0 |  |  |  |  |  |  |  | 24 | 7 | 3 | 6 | 6 |  |  |  |  |  |  |  | 24 | 0 | 1 | 0 | 3 |
|  | ß-TCP |  |  |  |  |  |  | 25 | 0 | 0 | 0 | 0 |  |  |  |  |  |  |  | 25 | 4 | 2 | 6 | 3 |  |  |  |  |  |  |  | 25 | 0 | 0 | 0 | 1 |
|  | ß-TCP |  |  |  |  |  |  | 26 | 0 | 0 | 0 | 0 |  |  |  |  |  |  |  | 26 | 4 | 2 | 4 | 4 |  |  |  |  |  |  |  | 26 | 1 | 0 | 2 | 1 |
|  |  |  |  |  |  |  |  |  |  |  |  |  |  |  |  |  |  |  |  |  |  |  |  |  |  |  |  |  |  |  |  |  |  |  |  |  |
| 5 | ß-TCP |  |  |  |  |  |  | 15 | 1 | 0 | 0 | 0 |  |  |  |  |  |  |  | 15 | 5 | 3 | 3 | 2 |  |  |  |  |  |  |  | 15 | 0 | 1 | 0 | 0 |
|  | ß-TCP |  |  |  |  |  |  | 16 | 0 | 1 | 0 | 0 |  |  |  |  |  |  |  | 16 | 5 | 3 | 5 | 3 |  |  |  |  |  |  |  | 16 | 2 | 1 | 1 | 1 |
|  |  |  |  |  |  |  |  |  |  |  |  |  |  |  |  |  |  |  |  |  |  |  |  |  |  |  |  |  |  |  |  |  |  |  |  |  |
| 6 | BCP | 24 | 0 | 0 | 0 | 0 |  | 14 | 0 | 0 | 0 | 0 |  | 24 | 3 | 2 | 2 | 3 |  | 14 | 5 | 2 | 3 | 3 |  | 24 | 0 | 1 | 0 | 1 |  | 14 | 1 | 0 | 0 | 1 |
|  | BCP | 26 | 0 | 0 | 0 | 0 |  | 15 | 0 | 0 | 0 | 0 |  | 26 | 4 | 2 | 4 | 3 |  | 15 | 5 | 3 | 4 | 3 |  | 26 | 0 | 0 | 1 | 1 |  | 15 | 1 | 0 | 0 | 1 |
|  | BCP |  |  |  |  |  |  | 16 | 0 | 0 | 0 | 0 |  |  |  |  |  |  |  | 16 | 4 | 3 | 3 | 2 |  |  |  |  |  |  |  | 16 | 0 | 0 | 0 | 0 |
|  |  |  |  |  |  |  |  |  |  |  |  |  |  |  |  |  |  |  |  |  |  |  |  |  |  |  |  |  |  |  |  |  |  |  |  |  |
| 7 | BCP | 25 | 0 | 0 | 1 | 0 |  | 15 | 2 | 1 | 0 | 0 |  | 25 | 5 | 3 | 5 | 4 |  | 15 | 4 | 2 | 3 | 3 |  | 25 | 2 | 0 | 0 | 1 |  | 15 | 0 | 0 | 1 | 0 |
|  | BCP | 26 | 1 | 0 | 1 | 0 |  | 16 | 0 | 0 | 1 | 0 |  | 26 | 5 | 3 | 5 | 5 |  | 16 | 5 | 2 | 6 | 3 |  | 26 | 2 | 0 | 0 | 1 |  | 16 | 1 | 0 | 1 | 0 |
|  | BCP | 27 | 0 | 0 | 0 | 0 |  | 17 | 0 | 0 | 0 | 0 |  | 27 | 9 | 8 | 9 | 7 |  | 17 | 7 | 3 | 5 | 4 |  | 27 | 2 | 3 | 3 | 3 |  | 17 | 2 | 1 | 1 | 0 |
|  |  |  |  |  |  |  |  |  |  |  |  |  |  |  |  |  |  |  |  |  |  |  |  |  |  |  |  |  |  |  |  |  |  |  |  |  |
| 8 | BCP |  |  |  |  |  |  | 14 | 0 | 0 | 0 | 0 |  |  |  |  |  |  |  | 14 | 5 | 3 | 3 | 3 |  |  |  |  |  |  |  | 14 | 0 | 1 | 0 | 0 |
|  | BCP |  |  |  |  |  |  | 15 | 0 | 0 | 0 | 0 |  |  |  |  |  |  |  | 15 | 5 | 3 | 3 | 3 |  |  |  |  |  |  |  | 15 | 0 | 0 | 0 | 1 |
|  | BCP |  |  |  |  |  |  | 16 | 0 | 0 | 0 | 0 |  |  |  |  |  |  |  | 16 | 5 | 5 | 3 | 4 |  |  |  |  |  |  |  | 16 | 1 | 1 | 0 | 1 |
|  |  |  |  |  |  |  |  |  |  |  |  |  |  |  |  |  |  |  |  |  |  |  |  |  |  |  |  |  |  |  |  |  |  |  |  |  |
| 9 | BCP |  |  |  |  |  |  | 23 | 0 | 0 | 1 | 0 |  |  |  |  |  |  |  | 23 | 3 | 3 | 4 | 2 |  |  |  |  |  |  |  | 23 | 0 | 0 | 0 | 0 |
|  | BCP |  |  |  |  |  |  | 25 | 0 | 0 | 1 | 0 |  |  |  |  |  |  |  | 25 | 5 | 2 | 3 | 2 |  |  |  |  |  |  |  | 25 | 1 | 0 | 1 | 0 |
|  | BCP |  |  |  |  |  |  | 26 | 0 | 0 | 1 | 0 |  |  |  |  |  |  |  | 26 | 4 | 2 | 4 | 5 |  |  |  |  |  |  |  | 26 | 1 | 0 | 0 | 0 |
|  |  |  |  |  |  |  |  |  |  |  |  |  |  |  |  |  |  |  |  |  |  |  |  |  |  |  |  |  |  |  |  |  |  |  |  |  |
| 10 | BCP | 25 | 0 | 0 | 0 | 0 |  | 15 | 0 | 1 | 0 | 0 |  | 25 | 6 | 3 | 3 | 3 |  | 15 | 5 | 3 | 3 | 4 |  | 25 | 1 | 1 | 0 | 0 |  | 15 | 1 | 1 | 0 | 2 |
|  | BCP | 26 | 0 | 1 | 0 | 0 |  | 16 | 0 | 1 | 0 | 0 |  | 26 | 3 | 2 | 4 | 5 |  | 16 | 3 | 2 | 4 | 3 |  | 26 | 1 | 1 | 0 | 2 |  | 16 | 1 | 1 | 1 | 1 |

For each implant the highest value of plaque index, sulcus bleeding index, and probing depth were scored. Plaque index was determined at the mesial, buccal, distal, and palatal implant surface: score 0: no plaque detected; score 1: plaque only recognized by running a probe across the smooth marginal implant surface; Score 2: plaque was seen by the naked eye; score 3: abundance of soft matter. Probing depth (mm) was determined at the mesial, buccal, distal, and palatal implant surface. Sulcus bleeding index was determined at the mesial, buccal, distal, and palatal implant surface: score 0: no bleeding when a periodontal probe was passed along the gingival margin adjacent to the implant; score 1: isolated bleeding spot visible; score 2: blood formed a confluent red line on margin; score 3: heavy or profuse bleeding. P-value: is based on Mann-Whitney U-test. SVF, stromal vascular fraction; ß-TCP, ß-tricalcium phosphate; BCP, biphasic calcium phosphates; Control, control side; Study, study side; n, total number of implants.
