# Supplementary material for: Long-Term Safety of Bone Regeneration Using Autologous Stromal Vascular Fraction and Calcium Phosphate Ceramics: A 10-Year Prospective Cohort Study
Source: Stem Cells Transl Med. 2023 Aug 1;12(9):617–30. doi: 10.1093/stcltm/szad045 (PMC10502529; doi:10.1093/stcltm/szad045)
Supplement: szad045_suppl_Supplementary_Table_S5 [file szad045_suppl_supplementary_table_s5.docx]

**Table S5** Peri-implant health, peri-implantitis, and peri-mucositis around dental implants in ß-TCP and BCP-treated patients with and without SVF-supplementation at 10-year follow-up.

| Pt# | Control/study side | Graft  material | Dental implant positions |
| --- | --- | --- | --- |
| 1 | Control  Study | ß-TCP  ß-TCP | **14, 15, 16**  *24, 25, 26* |
| 2 | Control  Study | ß-TCP  ß-TCP | *24, 25, 26*  *14, 15, 16* |
| 3 | Control  Study | ß-TCP  ß-TCP | *14,* 15, 16  *25, 26, 27* |
| 4 | Study | ß-TCP | **24,** *25, 26* |
| 5 | Study | ß-TCP | *15, 16* |
| 6 | Control  Study | BCP  BCP | *24, 26*  *14, 15, 16* |
| 7 | Control  Study | BCP  BCP | *25, 26,* **27**  *15, 16,* **17** |
| 8 | Study | BCP | *14, 15, 16* |
| 9 | Study | BCP | 23*, 25, 26* |
| 10 | Control  Study | BCP  BCP | *25, 26*  *15, 16* |

Peri-implant health, peri-implantitis, and peri-mucositis at 10-year follow-up. The control side was treated with only a calcium phosphate bone substitute, and the study wide was treated with a calcium phosphate bone substitute with SVF-supplementation. The dental implant positions are given according to the Fédération Dentaire Internationale system. Peri-implant health, underline; Peri-implantitis, bold; Peri-mucositis, italics. SVF, stromal vascular fraction; ß-TCP, ß-tricalcium phosphate; BCP, biphasic calcium phosphate; Pt#, patient number.
